# Spatial and temporal variability of the acoustic repertoire of Antarctic minke whales (*Balaenoptera bonaerensis*) in the Weddell Sea

**DOI:** 10.1038/s41598-023-38793-4

**Published:** 2023-07-22

**Authors:** Diego Filún, Ilse van Opzeeland

**Affiliations:** 1 Ocean Acoustics Lab, Alfred Wegener Institute for Polar and Marine Research, 27570 Bremerhaven, Germany; 2 Centro FONDAP-de Investigación en Dinámica de Ecosistemas Marinos de Altas Latitudes (IDEAL), Valdivia, Chile; 3 Helmholtz Institute for Functional Marine Biodiversity (HIFMB), Carl von Ossietzky University, 26129 Oldenburg, Germany

**Keywords:** Behavioural ecology, Ecology, Population dynamics

## Abstract

Since the attribution of the bio-duck call to Antarctic minke whales (AMW *Balaenoptera bonaerensis*), different studies have retrospectively identified several bio-duck call types at various sites throughout the Southern Hemisphere. The function of their vocal behavior however, remains largely unknown. Further insights into their repertoire usage may help to reveal the function of their calls. Here, we use passive acoustic monitoring (PAM) data collected across six locations throughout the Weddell Sea (WS) in 2013 and from PALAOA Station (Ekström Ice Shelf, eastern WS) in 2015, 2016 and 2017. In 2013, we detected 11 bio-duck call types throughout the WS between May and December, with additional acoustic activity in February on the western recorder AMW calls fell into four general call clusters. Seasonal patterns of calls showed variability between locations and years. Furthermore, this is the first study to show that similar to other baleen whale species, AMWs also produce *songs*.

## Introduction

Scientific knowledge on the fact that baleen whales produce sound is relatively recent; only since the 1940’s, the scientific community has become aware that baleen whales are not mute, but produce a large variety of sounds (e.g., Schevill and Watkins^1^). Insights into their repertoire and repertoire usage are fundamental for passive acoustic monitoring (PAM)-based remote sensing studies to assign calls to species and behaviors with certainty^2^. Baseline knowledge on acoustic behavior can nevertheless be difficult to obtain due to the logistic constraints to accessing the remote habitats that some species occupy during the time they are acoustically active^3,4^. Although the repertoire of the majority of baleen whale species has at least to some extent been described in literature, new studies attributing previously unknown acoustic signatures to certain species still continue to be published^5,6^. The Antarctic minke whale (AMW) bio-duck forms one of the best-known examples of an unknown signature which source was long an unsolved mystery^5^. First described and named by submarine personnel in the 1960s, ﻿the bio-duck had since been recorded at various locations in the Southern Hemisphere across lower to higher latitudes. Based on data derived from acoustic tags deployed on AMWs, the bio-duck sound was attributed to this species^5^ and existing recordings from the Southern Ocean (SO) could subsequently be explored for AMW acoustic presence^3,7^. The bio-duck consists of a regular series of downswept pulses, ranging from 50 to 300 Hz, with harmonics of up to 2 kHz^3,5,7^. In addition, AMWs are also known to produce single downsweep calls^8^. Bio-duck calls have been found to occur across lower, middle and higher latitudes in the SO and have been found to be present in these areas exclusively during austral winter and spring^3,6,9–11^. This seasonality distinguishes AMW calling behavior from the other baleen whale species, which also for the larger part migrate between lower latitude breeding and higher latitude feeding areas^12–14^. In the SO, other baleen whale species show increased calling during the austral summer season (but see^15–18^) during which time they are believed to use the area for feeding^12,13^. Across all locations where AMWs have been recorded to date, acoustic activity has been found strongly seasonal, peaking during the austral winter period^3,7,10,19^. Based on its coinciding timing with AMW female receptivity, acoustic behavior has beenAMW acoustic behavior has been suggested to potentially play a role in the context of reproduction^3,7^, but hypotheses on its behavioral function remain highly speculative as its occurrence coincides with female receptivity, but hypotheses on its behavioral function remain highly speculative. ﻿Although the number of studies on AMW vocal behavior is still limited to date, all report the existence of different types of bio-duck calls^5,7,9,11,20^. In absence of a systematically sampled data set, it nevertheless remains unclear how this call diversity relates to geographic and temporal distance in data collection.

In order to systematically explore the spatio-temporal patterns in AMW bio-duck call occurrence, we use two different passive acoustic data sets: (1) six moored positions deployed across the WS collected continuous data during 2013 and (2) three consecutive years of acoustic data collected from the PerenniAL Acoustic Observatory in the Antarctic Ocean, PALAOA, located in the Ekström Ice Shelf, Eastern WS^21^.

## Methods

For this study, we used data from autonomous passive acoustic recorders, deployed and collected during several expeditions with the *RV Polarstern* at different sites in the WS.

### Weddell Sea data collection

**Figure 1:**
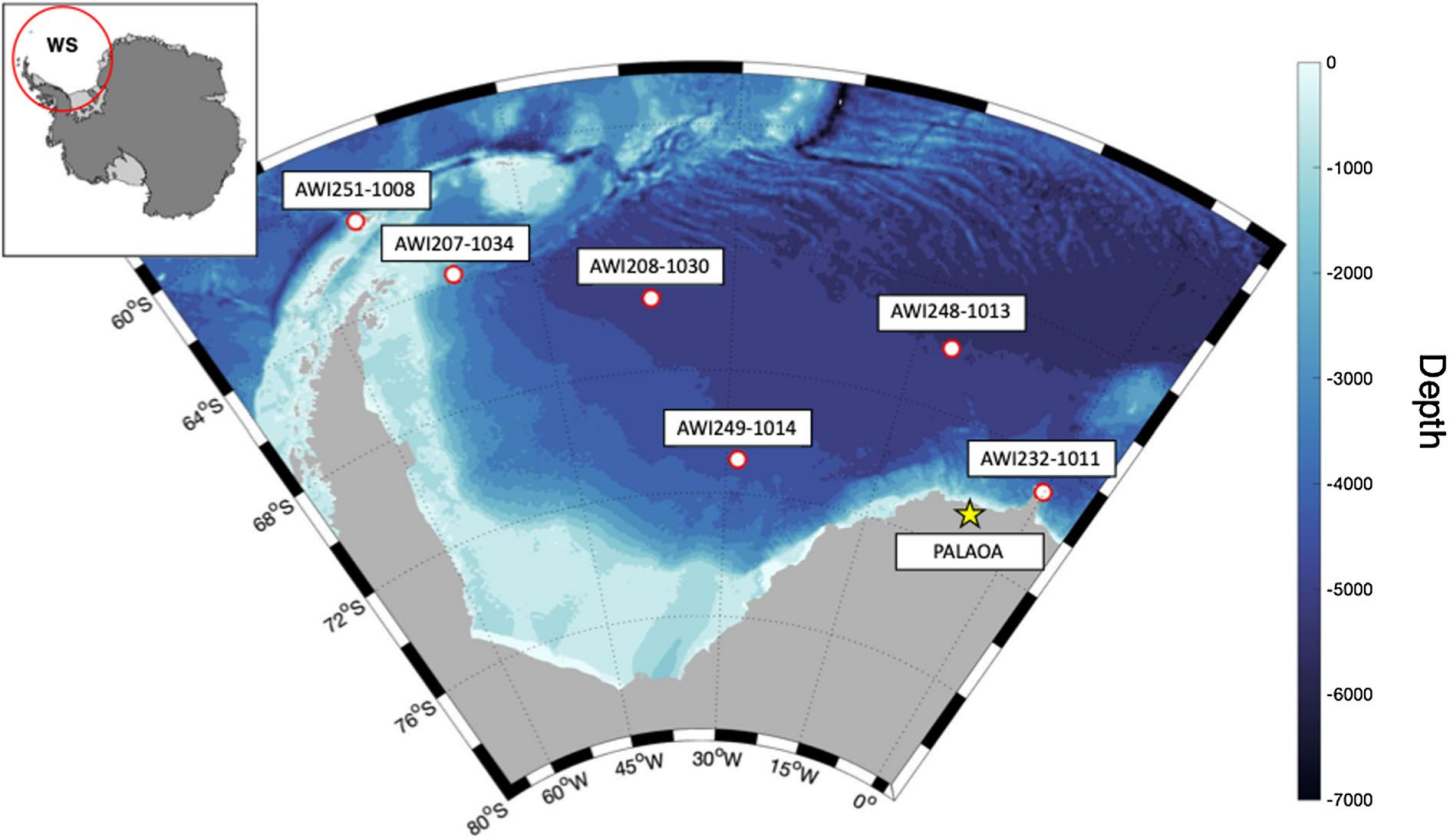
Map of the Weddell Sea area showing the locations where passive acoustic recordings were collected. Red-white dots represent the offshore mooring positions across the Weddell Sea from which one year (2013) of recordings was used. The yellow star represents the location of the PALAOA observatory on the edge of the Ekström ice-shelf from which three years (2015–2017) of consecutive recordings were used. Map was generated with M-MAP in MATLAB^71^.

The Weddell Sea (WS) data set was collected using hydrophones forming part of the HAFOS (Hybrid Antarctic Float Observation System) network of oceanographic deep-sea moorings deployed throughout the Antarctic Weddell Sea^22^. For this study, data were used from 2013, from six different mooring positions distributed between 59° S and 69° S and from 0° W to 56° W across the Weddell Sea (Fig. 1, Table 1). Passive autonomous acoustic recorders (SonoVaults developed by Develogic GmbH, Hamburg) were programmed to record continuously with a sampling rate of 5333 Hz at 24-bit (TC4037-3 RESON hydrophone with a nominal sensitivity of approximately – 193 dB re 1 V/µPa^3,22,23^).

**Table 1:** Location, sampling rate, duty-cycle and recording data of passive acoustic recorders.

Mooring ID	Latitude	Longitude	Sampling rate (kHz)	Effort (days)	Acoustic detections	
					Days	Hours
AWI251-1008	−61.015	−55.827	5.333	291	30	201
AWI207-1034	−63.701	−50.827	5.333	281	84	749
AWI208-1030	−65.621	−36.422	5.333	289	101	978
AWI249-1014	−70.893	−28.891	5.333	305	75	531
AWI248-1013	−65.621	−36.422	5.333	316	96	748
AWI232-1011	−65.998	−0.109	5.333	269	96	718
PALAOA2015	−70.500	−8.217	96.000	365	97	736
PALAOA2016	−70.500	−8.217	96.000	365	105	893
PALAOA2017	−70.500	−8.217	96.000	270	112	977

### PALAOA data collection

The PerenniAL Acoustic Observatory in the Antarctic Ocean (PALAOA) is a stationary recording location positioned on the Ekström Ice Shelf at 70° 30.717' S, 008° 13.178' W (Fig. 1). It collects continuous underwater recordings underneath the ice shelf edge with hydrophones deployed through boreholes^24^. During 2014, the observatory switched from two-hydrophone, live streaming mode^21^, to archival data collection with a single hydrophone. Here, we used data from 2015 to 2018, which were collected using the archival set up. Recordings are made continuously year-round with a RESON TC4032 hydrophone (5 Hz to 120 kHz, sensitivity –170 dB re 1 V μPa^–1^) deployed underneath the 100 m thick floating Antarctic ice shelf^21^. Water depth below the floating ice shelf is approximately 160 m. The hydrophone is at a depth of 80 m below the floating ice shelf. The hydrophone is connected to a SonoVault unit sensitivity −133.4  ± 1 dB, sampling rate 96 kHz, 24-bit resolution)^24^ that is positioned on the ice in a protective case. It records the data and stores it on SD cards. For the subsequent analysis process, the PALAOA data were decimated to 6,000 Hz (Table 1).

### Manual data preprocessing

**Figure 2:**
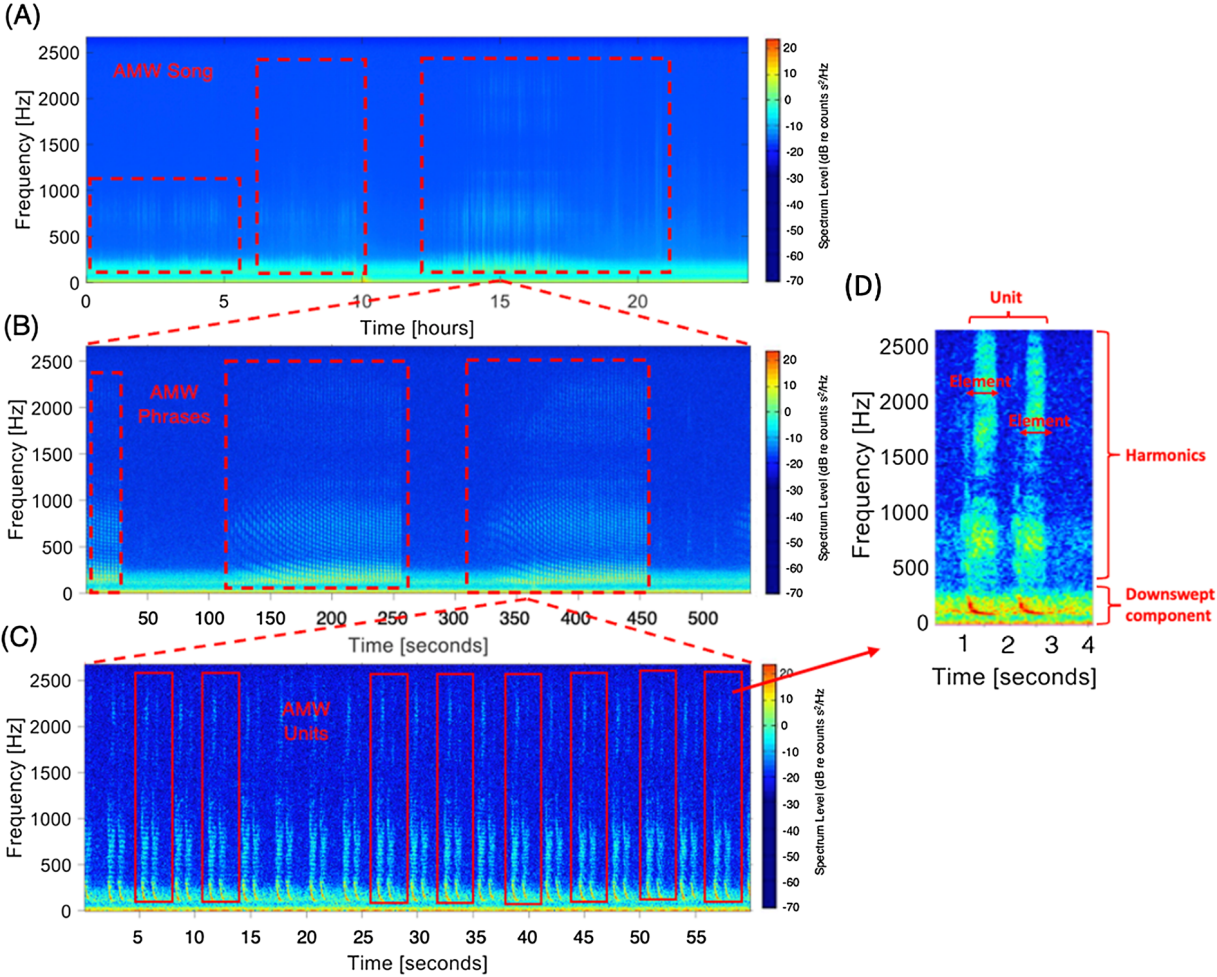
Spectrograms with examples of AMW (**A**) Song bouts, (**B**) phrases, (**C**) bio-duck units consisting of the single elements or pulses and (**D**) spectrogram with the different parts that compose a bio-duck call. Spectrogram (**B**) exhibits a Lloyd Mirror effect in the AMWs phrases. The visualized data are from the position AWI208-1030 during the 15th September 2013.

To detect the occurrence of AMW bio-duck calls in the recordings, all nine data sets of acoustic recordings were manually processed using the Matlab-based (Mathworks, Natick, MA) custom-written software program Triton^25^ to create long-term spectral averages (LTSAs). LTSAs visually represent time series of averaged spectra (Fig. 2). The successive spectra for all the data were calculated by averaging 60 s of acoustic data with a frequency resolution of 1 Hz. The LTSAs were analyzed with a window length of 12 h to identify AMW acoustic signatures. When presumed AMW bio-duck calls were observed in the LTSAs, a 30-s spectrogram window (overlap = 90%, FFT = 1050) of that time section was inspected visually and aurally to verify the presence of AMW bio-duck calls^3^. Here, a bio-duck call, hereinafter also referred to as bio-duck unit, was defined as a series of clustered downswept pulses, with single pulses separated by < 1-s^7,11^. Generally, bio-duck units never occurred alone, but always occurred in sequences (Fig. 2)^3^. AMW acoustic presence (i.e., AMW positive hours) was assessed on an hourly basis for all acoustic data sets. Hours during which a sequence of at least 1 min of consecutive bio-duck units was present were considered AMW positive. For all AMW bio-duck sequences, a 10-s audio fragment was extracted for further subsequent detailed analyses and classification into different bio-duck unit types.

### Detailed analysis of bio-duck unit types

Prior to automated feature extraction for bio-duck unit classification, all 10-s fragments that were extracted from the LTSAs were preprocessed to optimize computations. Spectrograms (1,125-point FFT, 85% overlap, Hanning window) of the 10-s audio fragments were manually checked in RStudio Version 1.2.5042 with the ‘Seewave’ package^26^ to corroborate the quality of the signals. To the 10-s audio fragment an amplitude filter of 20 dB was applied to increase the SNR of the bio-duck unit spectrum. Furthermore, the 10-s audio files were frequency filtered between 40 and 500 Hz and the signal-to-noise ratio (SNR) of bio-duck units in the clip was calculated. In cases for which multiple bio-duck units were detected in the 10-s fragment, the unit with the highest SNR was selected for further processing. To define SNR, for each bio-duck unit, background noise level was measured 0.6 s prior to the detected bio-duck unit event in the frequency bands between 40 and 500 Hz. Signal level was measured 1.1 s after the time point of background noise measurement. Only bio-duck units with a resulting SNR that was sufficiently high (i.e., exhibited a clearly higher-energy signal of interest in contrast to background noise, > 10 dB SNR), were selected for further processing using a pulse detector^27^. The pulse detector was used to automatically extract call characteristics from each bio-duck unit for the classification and was designed to work using the low frequency downswept component present in all bio-duck units (Fig. 2). Bio-duck pulse detection thresholds were custom-defined based on the individual bio-duck unit SNR (SNR = 20*log10(rms_(signal)_/rms_(noise)_). For acoustic detections with a SNR < 5 dB, we applied a detector threshold = 50, for detection with SNR > 5 dB and SNR < 10 dB a threshold = 30, with SNR > 10 dB and SNR < 15 dB threshold = 20, with SNR > 15 dB and SNR < 20 dB threshold = 15 and with an SNR > 20 dB a threshold = 12 (Appendix Fig. 2). To reduce false positives of other pulsed signals potentially present in the 10-s fragments, only detections of pulses with a duration within the range of 0.055–0.9 s (which were the known minimal and maximal durations of AMW pulses based on manual measurements on a subset of the data, Appendix Table 1) were included as part of the bio-duck pulse sequence (Appendix Table 2).

For the final classification of the different AMW units detected in our data set, we used the automatically detected elements to extract different variables with a custom-built algorithm in RStudio Version 1.2.5042, using the R packages ‘Seewave’^28^ and ‘warbleR’^27^.

Parameters that were automatically extracted by the algorithm included the number of pulses (NP), total duration (TD), mean inter-pulse interval (IPI), duration first pulse (DFP), duration last pulse (DLP) and peak frequency (PF) (Appendix Table 1 & Appendix Fig. 1). The algorithm is based on an amplitude detector capable to automatically extract and calculate different measurements from the waveform of AMW bio-duck units between 40 and 500 Hz. Although the different types of bio-duck can have harmonics up to 2000 Hz, these are not a reliable feature as their presence and quality depends on different factors such as, for example, proximity of the animal to the hydrophone, ambient noise levels and sound directionality. Only the fundamental frequency of the downswept pulses was included in the measurements as robust frequency parameter.

We separated the different bio-duck units using cluster analysis based on the calculated acoustic parameters in order to optimize the classification. The different parameters extracted from each bio-duck unit type were used to perform a hierarchical agglomerative cluster analysis to generate unsupervised unit type groups. A Euclidean method was used to calculate the distances between the different clusters. To determine the number of clusters (*k*), we used the ‘NbClust’ package^29^. This automatically calculates and provides 30 different indices for determining an appropriate number of *k* from the different results obtained by varying all combinations for number of clusters, distance measures and clustering methods.

### Monthly acoustic variability

The rate of occurrence of the different AMW units was calculated as the ratio of the sum of hours over which each type of unit was present and the total number of hours with detections for each month.

### Sea ice concentration

The values of sea ice concentration for this study were extracted from (1) a combination of satellite sensor data from the Nimbus-7 Scanning Multichannel Microwave Radiometer (SMMR), (2) the Defense Meteorological Satellite Program (DMSP) -F8, -F11 and -F13 Special Sensor Microwave/Im rs (SSM/Is), (3) the DMSP-F17 Special Sensor Microwave Imager/Sounder (SSMIS), with a grid size of 25 km and (4) the satellite images from the Advanced Microwave Scanning Radiometer for EOS (AMSR-E) satellite sensor with a grid size of 6.25 km. These data were used to calculate the daily sea-ice concentration of the area within 40 km radius around each recording location deployed throughout the Weddell Sea^30,31^ to explore the correlation between AMW acoustic occurrence and local sea ice concentration.

### Calculation sunset-sunrise diel pattern

To calculate the hours of light and darkness in a day and relate these to AMW vocal behavior, we used the Sunrise/Sunset tool in MATLAB^32^*.* We calculated the equation of time and sun angle to estimate the periods of sunrise and sunset in the UTC time zone according the specific latitude and longitude for every recording position. The calculations were based on data available from NOAA’s Earth System Research Laboratory. The values were used to identify diel patterns of AMWs acoustic occurrence at the different monitoring positions.

## Results

### AMW hourly acoustic presence

In both the WS and PALAOA data sets, bio-duck units occurred from May to November. The duration of the period during which bio-duck calls were recorded however varied between recording locations from 4 to 6 months; with longer acoustic presence occurring at higher latitudes^3^. AWI207-1034 was the only position that showed AMW acoustic activity during January and February.

For the WS data, a total of 1751 days in 2013 from six different anchoring positions were analyzed to detect and classify AMW bio-duck signatures. The number of days with AMW acoustic presence varied between sites, with lowest AMW presence at site AWI251-1008 (201 h on 30 days; Table 1) and most AMW acoustic presence at AWI208-1030 (978 h on 101 days; Table 1).

For the PALAOA data set, a total of 937 days from the 3-year period spanning 2015 until 2018 (Table 1) were analyzed. In 2015, we detected AMWs on 97 days corresponding to 736 h with bio-ducks. In 2016, the number of days with AMW acoustic presence was 105 with a total of 893 h. In 2017, AMWs were detected on 112 days, corresponding to 977 h of bio-duck presence.

### Bio-duck unit type classification

**Figure 3:**
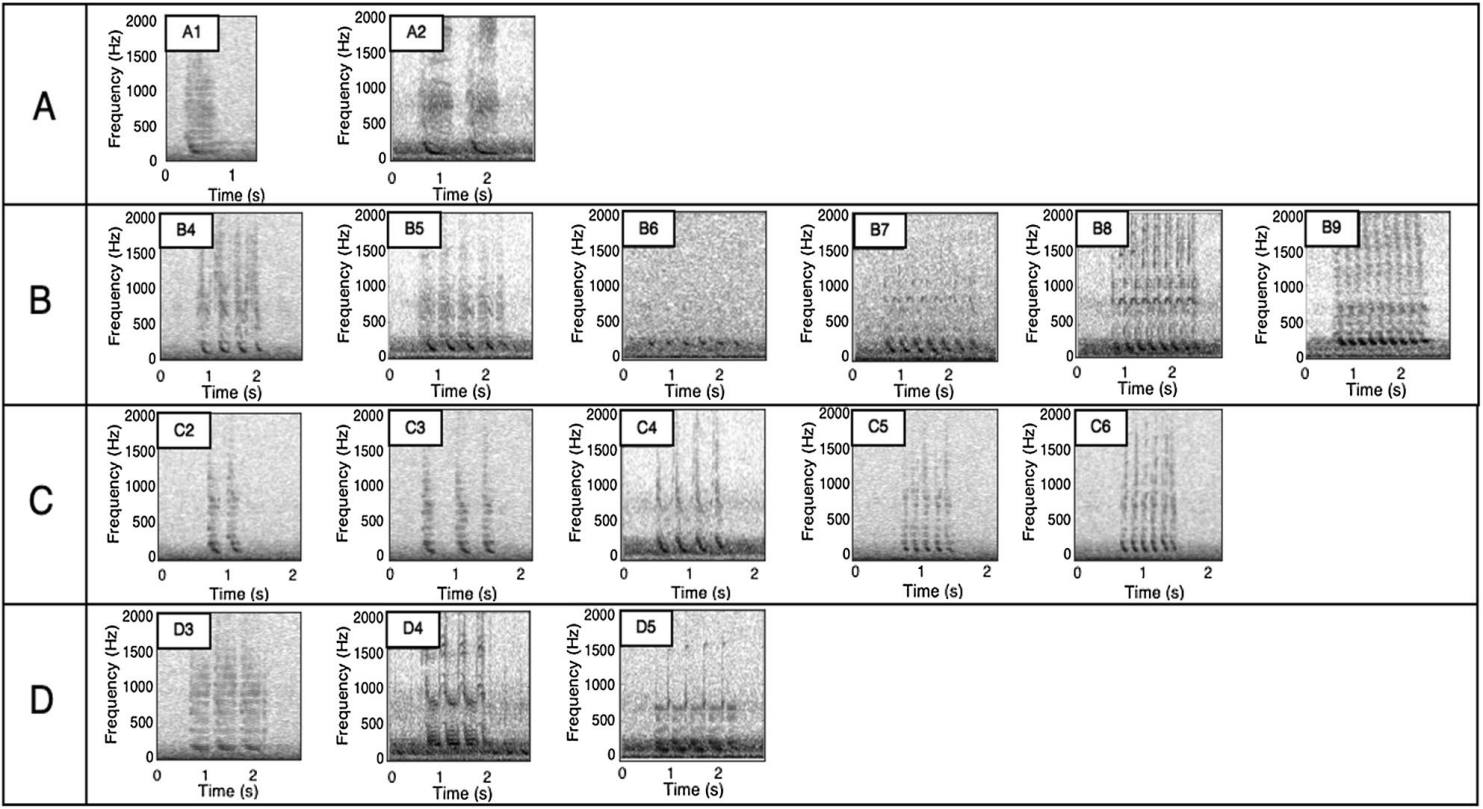
Spectrograms of the different AMW unit types detected and classified from the HAFOS (2013) and PALAOA data (2015–2017). Spectrogram settings HAFOS data: fast Fourier transform (FFT) length of 1125 samples, 95% overlap and Hanning window.

**Figure 4:**
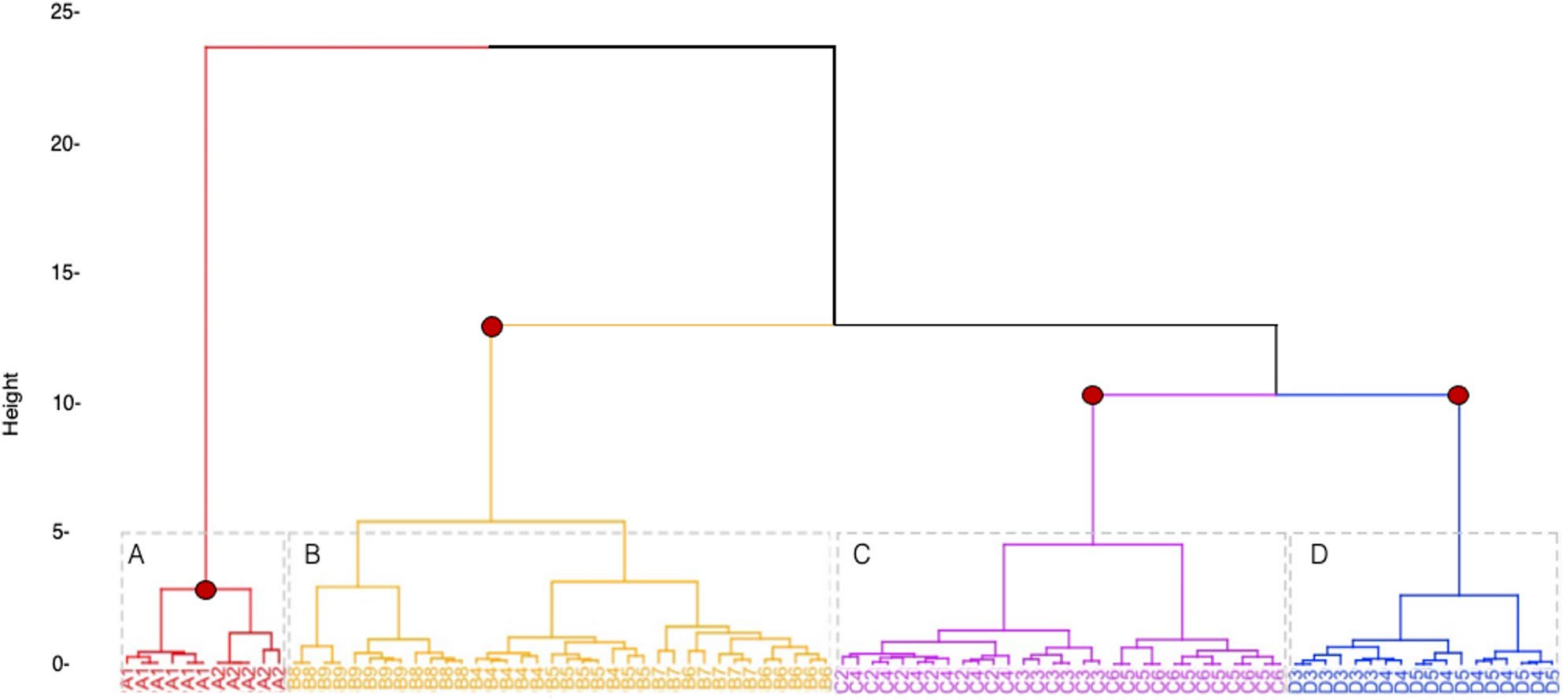
Hierchical dendrogram. Visualization of the different bio-duck unit classification groups detected in the HAFOS network (Weddell Sea) and PALAOA. NP, TD, DFP, DLP, IPI and PF variables used to split. Dots in red represent clusters with AU larger than 95%. Each gray rectangle represents the different categories (**A–D**).

Bio-duck units typically consisted of sequences of 1–9 pulses with an IPI between 0.03 and 0.40 s. The duration of the detected single bio-duck elements ranged from 1.02 to 1.90 s (Appendix Table 2). The durations of the first and last pulse of a bio-duck sequence were included as variables in the classification and measured 0.09–0.71 s and 0.05–0.42 s, respectively. The peak frequency of the downswept bio-duck element varied substantially between the different bio-duck unit types and ranged from 114.5 to 197.9 Hz (Appendix Table 2). Based on the extracted measurements (Appendix Table 2), the cluster analysis classified the 16 different bio-duck unit types into four groups (Groups A-D) (Figs. 3 and 4). Group A comprises two types “A1” and “A2.” Group B is composed of six types; “B4”, “B5”, “B6”, “B7”, “B8” and “B9”. Group C has five types; “C2”, “C3”, “C4”, “C5” and “C6”. The last group D is composed of three types; “D3”, “D4” and “D5” (Figs. 3 and 4).

### HAFOS data set: spatial variability in AMW call repertoire

**Figure 5:**
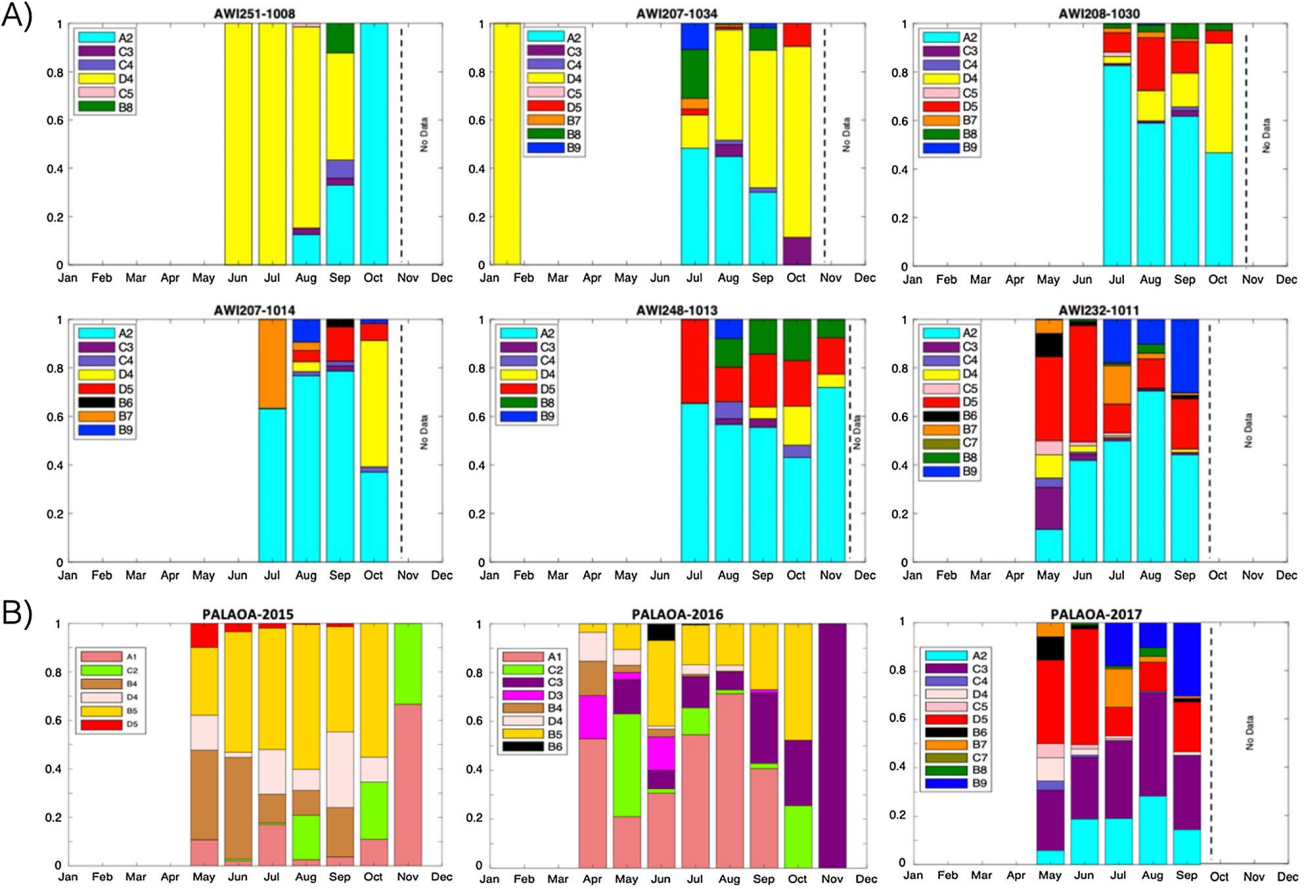
Proportion of occurrence of the different bio-duck unit types per month identified for (**A**) 2013 from the 6 HAFOS sites and (**B**) from the multi-year PALAOA data. The dotted line indicates periods during which no data were collected.

The occurrence of the different bio-duck unit types varied between HAFOS recording positions. Position AWI251-1008 exhibited least variability in recorded AMW vocalizations and only six types of bio-ducks were recorded at this location (Fig. 5). At position AWI232-1011, 11 different types of bio-ducks were recorded, showing the richest AMW repertoire of all recording sites. At positions AWI207-1034 and AWI208-1030, nine different types of bio-duck were detected, whereas at positions AWI 249-1014 and AWI 246-1013, eight and seven call types were detected, respectively (Fig. 5).

Bio-duck type “A2” and “D4” were detected at all HAFOS positions in 2013 (Fig. 5). In addition, the “D4” type was detected during January (on 3 days) at position AWI201-1034. This position was the only one to record AMW acoustic activity during the summer period in the Weddell Sea area. Bio-duck types “C4”, “D5”, “C3” and “B9” were recorded at all positions, except for AWI251-1008. Unit type “B8” was found at all positions, except at AWI20-1014.

At positions AWI251-1008 and AWI207-1034, type “D4” was the most dominant and the call “A2” the second most occurring type (Fig. 5). For the remaining positions, the most dominant was type “A2” with “D4” exhibiting the second most frequent occurrence at positions AWI20-1034, AWI208-1030 and AWI20-1014 and “D5” the second most common at positions AWI248-1013 and AWI232-1011 (Fig. 5). We detected only 4 h in our dataset with simultaneous presence of more than one bio-duck unit type.

### PALAOA data set: multi-year patterns in AMWs calling behavior

The PALAOA data enabled the comparison of AMW acoustic presence, repertoire composition and usage over a time span of 3 years. In 2015, we detected the acoustic occurrence of AMWs between May and November (Appendix Fig. 4) and six different bio-duck units were identified, being “A1”, “B4”, “B5”, “C2”, “C4” and “D5”. In 2016, AMWs were acoustically present between April and November and eight different bio-duck calls “A1”, “B4”, “B5”, “C2”, “C3”, “D3” and “D4” were found to be present (Fig. 5). During 2017, AMWs were found acoustically present between May and September and 11 different types of bio-ducks were identified: “A2”, “B6”, “B7”, “B8”, “B9”, “C3”, “C4”, “C5”, “C7”, “D4” and “D5” (Fig. 5). Due to electronic noise in the recordings, it was not possible to analyze the 2017 data collected between October and December.

During the 3 years of continuous monitoring, the proportion in which the different units were present was not constant. In 2015, the predominant unit was “B5” (47.4% of all detections). The secondary call was “B4” with 18.7% of all detections. In 2016, the predominant unit type was “A1”, corresponding a 42.8% of all detections that year. The secondary bio-duck was “B5” with a 21.1%. Finally in 2017, the most frequently detected unit was “C3”, composing 32.7% of all detections. The second most detected unit in that year was “D5” with a 22.3%, closely followed by the unit type “A2” was 18.8% (see Appendix Table 3).

### Diel AMW calling pattern

**Figure 6:**
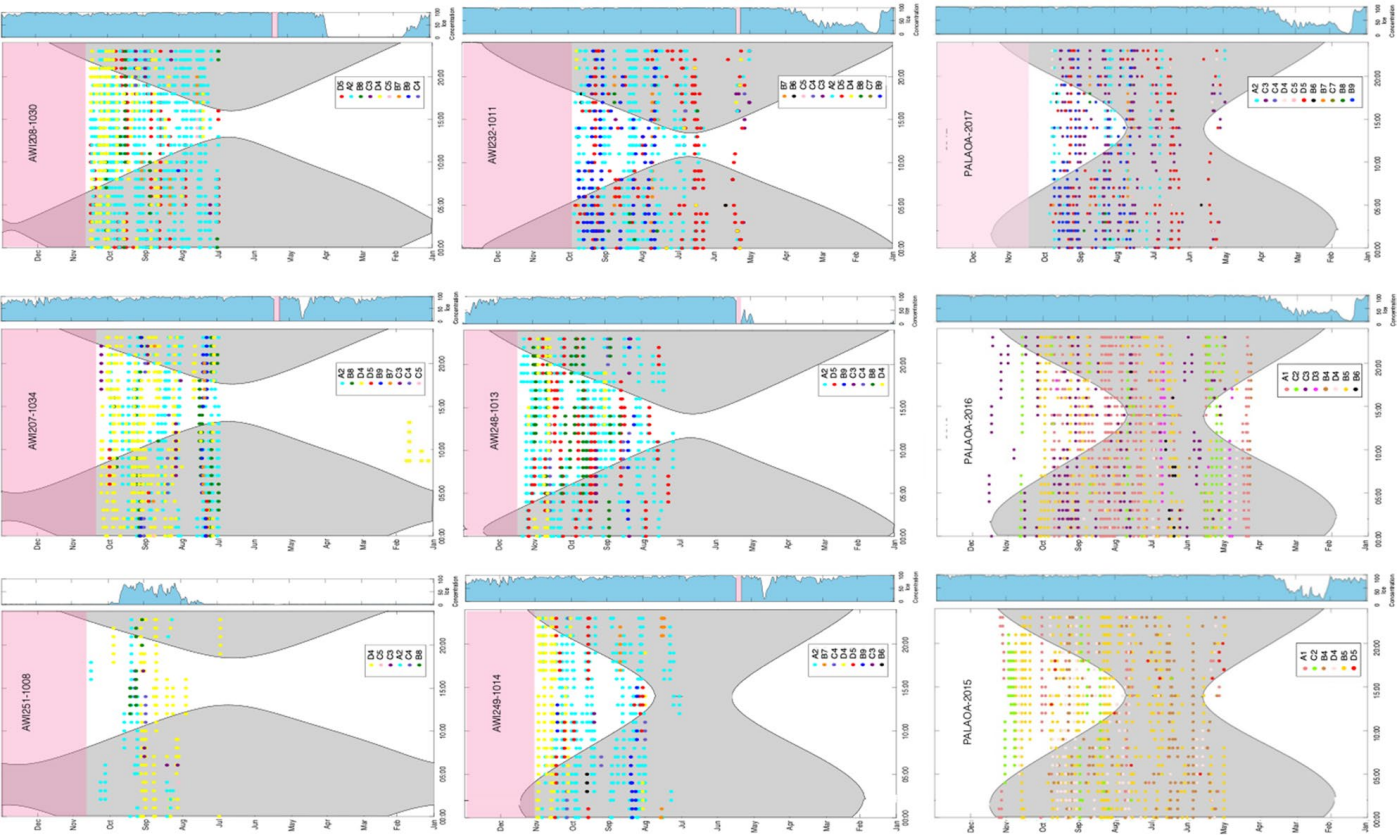
Seasonal and diel distribution of the different bio-duck unit types from the HAFOS data set during 2013 and the multi-year PALAOA data. Every dot represents an hour with AMW acoustic detection. The dot coloring represents the different AMW bio-duck unit types detected in every monitoring position. *Y*-axes shows date; left *X*-axis time of the day (hours); right *X*-axis ice concentration in a 50 km radius. Grey shading represents the periods between sunset and sunrise. Light pink shading represents periods during which no data were collected.

For both data sets, we did not find a daily pattern in AMW acoustic activity or unit type usage when acoustic occurrence was explored in relation to local light regimes across months (Fig. 6).

## Discussion

This study provides the first quantitative description and classification of the acoustic repertoire of AMWs in the Weddell Sea. Our classification method distinguished four clusters of bio-duck call types, each consisting of different subtypes of bio-duck units that all have a similar acoustic structure, but differ in the number of pulses and/or the duration of the inter-pulse interval. The systematic classification and nomenclature method applied in this study forms a critical step forward in understanding more about the acoustic behavior of AMWs. With an automatic classifier we can now systematically and objectively analyze how the acoustic repertoire of these whales is composed and how it varies over space and time.

Several of the bio-duck unit types that we identified in the Weddell Sea data have also been described by studies conducted at other locations, e.g., the West Antarctic Peninsula bio-duck ‘A1’ and ‘A2’ in Dominello and Širović^7^, resembles C4 and category B in our study. Also, ‘B’ and ‘C’ in Risch et al.^5^, exhibit similarities in shape to C5 and C3 in the Weddell Sea data. Furthermore, bio-duck units ‘A1’ and ‘A2’ recorded off South Africa^11^ seem to match our C4 and A2, while the Australian type ‘2B’^9^ resembles our A2. Overall, the AMW sounds described in earlier studies match our overall classification clusters A–D, but seem to represent additional bio-duck unit subtypes differing from our data in terms of variability in the number of pulses. An in-depth comparison of the geographic variability across bio-duck recordings from other studies was beyond the scope of the current analyses, but is in progress.

Although systematic large-scale comparisons are not yet available, the overall accordance between our classification groups and data from studies conducted in other latitudes and sectors of the Southern Ocean nevertheless demonstrates that the AMW acoustic repertoire overall has a consistent structure of bio-duck call composition. The existence of the bio-duck subtypes recorded simultaneously in the same region, may reflect differences in acoustic behavior between cohorts and the behavioral context in which calls are produced. It may also suggest the co-existence of sympatric acoustic populations. Furthermore, the observed temporal variability in bio-duck subtypes, shown by the PALAOA data, suggests that AMW calling behavior changes over the course of seasons, reminiscent of humpback whale (*Megaptera novaeangliae*) song evolution^33^.

### Do Antarctic minke whales sing?

In this study we postulate that, in analogy to the majority of other large whale species e.g., humpback whales^34^, right whales (*Eubalaena *sp.)^35^*,* fin whales (*B. physalus*)^36^ and blue whales *(B. musculus*)^37^, the AMW bio-duck unit sequences fit the definition of song (e.g., Cholewiak et al.^38^. AMW bio-duck calls can be broken down into “elements”, “units” and “phrases” that are repeated in a regular pattern, forming song bouts as has been described for the other mysticete species^39–41^. Furthermore, the existence of themes (i.e., a structured repetition of phrases) cannot be excluded—preliminary observations in our data showed that on seven occasions at four of the six WS monitoring positions, sequences of bio-duck units that were apparently produced by one individual, alternated between different bio-duck types within the same sequence (Appendix Fig. 2). This behavior suggests that individuals are able to vary bio-duck units within a phrase.

In other baleen whale species, the overall structure of songs remains constant over many years, even decades^42,43^. In comparison with AMW bio-duck calls, the function of pulse trains emitted by North Atlantic Minke Whales (NAMWs), (*B. acutorostrata*), is similarly unknown. However, for NAMWs differences in acoustic repertoire have also been found along latitudinal and longitudinal gradients^44,45^. Some observed differences in call type distribution could indicate a shift from summer feeding activity at higher latitudes to presumed winter breeding at lower latitudes^46,47^. In comparison with AMW data reported here, NAMW vocalisations demonstrate both similarities and differences in dominant call structure between recording locations (e.g. similarities at NE Atlantic and central NW Atlantic locations^44,45,48^; structural differences between calls recorded at Stellwagen Bank National Marine Sanctuary (SBNMS) in NW Atlantic and the Caribbean^47,49^. However, for both NAMWs and AMWs further studies are needed to clarify geographical variation in call types and repertoire, and to explore their function in the context of minke whale behavioral ecology.

Our study suggests that AMWs produce songs of less complexity than described for humpback whales^34^. Their structure is more similar to blue and fin whale song, in which a few simple and highly stereotyped phrases are repeated to form a song^36,50,51^. The rate of change of AMW repertoire between regions resembles the sudden shift in fin whale songs that was observed in the northern Pacific Ocean^52^. In fin whales these shifts have to date been observed only rarely and have been suggested to follow shifts in the primary resident populations between areas. In AMWs however, the acoustic repertoire varies from year to year. Each year there is a predominant call, which is detected throughout the entire acoustic season. This call is present in > 40% of the total hours with bio-ducks records, but changes in type every year. Each year’s new dominant call seems to correspond to a bio-duck type that already started to increase in presence in the repertoire towards the end of the previous year. The dominant call of one year does not disappear the following year, but its occurrence decreases, becoming the second or third most frequent. The composition of the overall acoustic repertoire also changes dramatically from year to year: vocalizations that disappeared from one year to the next, and those that appeared for the first time, were generally detected at low levels of occurrence. These calls individually constituted < 10% of the total acoustic repertoire of the season. Based on our preliminary multi-year data analyses, our data suggest that, as in humpback whales, individual AMWs may change their repertoire from year to year^53^. ﻿This inter-annual change in AMWs acoustic repertoire, matches the pattern of song “revolutions” in humpback whales, where the single population-wide shared song type is rapidly replaced by a new, novel song type introduced from a neighboring population^54,55^. We speculate that the dynamics of AMW vocal behavior potentially reflect a similar mechanism through which the simultaneous presence of different groups or populations of AMWs mutually affects the composition of the annual repertoire.

### The function of the bio-duck call in AMWs

For AMWs, it remains unknown whether bio-duck sounds are produced by one or both sexes, hence until the singers are sexed, we can only speculate based on what is known from other singing baleen whale species. This comparative evidence suggests that the more elaborate vocal displays are most often produced males^36,56,57^. Females in these species are known to generally produce shorter and simpler calls that serve in e.g., mother-calf communication or are produced during migration^58^. For AMWs, the limited studies available seem consistent with the hypothesis that these call sequences may be produced by males. The observed acoustic behavior appears to coincide with the breeding season from May to November^59,60^. Also, the observation that there is no diel pattern in calling activity, matches observations in other species where mating-related calling behavior did not exhibit any light-regime related fluctuations (e.g., Van Opzeeland et al.^61^; Thomisch et al.^18^; Burkhardt et al.^62^). Furthermore, given the long duration (i.e., hours) of some of the bio-duck bouts encountered in this study (Fig. 2), is reminiscent of typical reproductive vocal display during which individuals are investing a substantial amount of time and energy in the production of extended bouts of acoustic activity^36,53^. These results add support to the hypothesis that the AMW bio-duck may function as a form of reproductive advertisement display that potentially communicates information on fitness of the caller. Clearly, further work is needed before further conclusions can be drawn on whether AWM calls are produced by one or both sexes.

### Geographic characterization and population identity

Previous work on minke whale acoustic behavior suggests that a simple downswept call is used across geographic regions by different minke whale sub-species (e.g., AMWs as well as NAMWs in NE Canada)^8,63^. Dwarf minke whales (another sub-spp), produce more complex vocalizations, such as the ‘star-wars’, or ‘boing’ sound, which are regionally distinctive, whereas their ‘thump train’ resembles the pulse train calls that have also been described for other minke whale subspecies from both northern and southern hemispheres^5,49,64^. ﻿Here, we show that bio-duck calls are also regionally distinctive and can potentially be used to identify different populations or stocks of AMWs in the Southern Hemisphere, in analogy to how blue whale acoustic signatures can be used to attribute animals to regional populations^37^.

Previous studies implementing different genetic techniques provide strong evidence to retain the hypothesis of multiple AMW stocks in the Southern Ocean^65^. These studies suggest that there are at least two stocks present in the SO: an eastern Indian (I) and a western South Pacific (P) stock^66^. These stocks are thought to mix across a soft boundary, which would probably best be placed near 165 °E^65^. These stocks may be related to the breeding areas proposed for the eastern Indian Ocean and western South Pacific. Although our acoustic data come from the Atlantic sector of the Southern Ocean, our results also indicate the presence of multiple clusters in the Weddell Sea sector (Appendix Fig. 2). There is one group in the west (Antarctic Peninsula region) and another in the east, closer to the Greenwich meridian area. In addition, there is a third group that could be in between these two groups which is where potential exchange between groups could take place, as has been described for humpback whales (Appendix Fig. 2)^24^.

We postulate two possible hypotheses regarding the acoustic behavior of AMWs in the Weddell Sea. The first is that the predominant bio-duck call types are associated with specific behavior performed by the animals in the respective area. If certain calls are linked to behavior that is area-specific, the observed differences in repertoire usage could reflect spatial variation in AMW habitat usage across the Weddell Sea. However, in this case it would have been more likely that the period during which calls occur, differs between recording locations, as presence and behavior of the animals may be linked to different habitat features that not all occur at the same time, forcing animals to move between sites^67^. Our second hypothesis therefore, is that these calls reflect the existence of different groups or subpopulations of AMWs that occur simultaneously during the winter and spring season in different parts of the Weddell Sea (Appendix Fig. 2). If the second hypothesis holds true, our findings for 2013, the year during which we had recordings for multiple sites, could indicate that three possible groups of AMWs coexist in the Weddell Sea: the group near the Antarctic Peninsula (sites AWI251-1008 and AWI207-1034) using the “D4” call as a distinctive call type, secondly the group present in the center of the Weddell Sea using the “A2” call as a distinctive call type (Appendix Table 3 and Appendix Fig. 2). And finally, the third group that exhibits “D5” as distinctive call and that would correspond to the area east of the Greenwich Meridian (during the year 2013). Call type “D5” was not often present in our data set, possibly because our Greenwich recorders were at the western limit of its distribution.

However, given that the multi-year data from PALAOA show that the AMW repertoire composition changes from year to year, single bio-duck call types may not be reliable indicators of stocks. Instead, call repertoires should be interpreted in the context of larger scale patterns in AMW vocal behavior, again, more analogous to how song is specific to humpback whale stocks.

## Conclusions

The function of the AMW bio-duck call is still unknown. It has been suggested that this call is related to mating behavior since it occurs mostly during the breeding season (during May until November)^60^. There are similarities in the structure of AMW bio-duck calling to the songs of other baleen whale species that are believed to be associated with reproductive displays, which supports our previous suggestion that bio-duck calls are also associated with mating behavior (Filun et al.^3^). The Weddell Sea (or any region of the Southern Ocean south of 60°) is nevertheless not a known breeding ground for AMWs, although this may also reflect the limitations of what is known on AMWs in their ice-dominated habitat. In other cetacean species, mating-related vocal behavior (with or without actual mating activities) has also been found to occur regularly on the feeding grounds^68^. According to the description of whale songs, our study shows that AMWs are capable of producing songs of medium complexity, which in some cases were found to be broadcasted for hours. Although the songs produced by AMWs are not as complex in structure as those of humpback whales or bowhead whales, we describe them as medium complex due to the AMW repertoire of sounds and the fact that the constitution of this repertoire can vary from site to site and year to year. We systematically documented a wide diversity of bio-duck calls, which can be classified into groups according to the structure of the pulses that constitute them. These groups are made up of different bio-duck call types that coincide in overall structure but differ in their number of pulses. Our methods have proved to be robust for multi-site, multi-year data and can therefore be applied to further data sets to enable consistent comparisons of additional data, covering larger spatial and temporal scales.

Finally, we postulated the potential distinction of groups or populations of AMWs based on the acoustic signals they emit, as has been shown for other whale species^37,52,69,70^. Future studies integrating more acoustic data, covering more spatial and even circumpolar coverage in the Southern Ocean are needed to test the hypothesis about the identification of different populations of AMWs based on their vocal behavior. Nevertheless, the combined spatial and temporal dynamicity of the AMW vocal behavior is likely to complicate identification based on single recordings. Along these lines, implementing and extending the area and timing of AMW monitoring effort in low latitude areas would help to better understand their distribution and identify potential migration routes. Finally and now lastly, research needs to focus on the larger scale pattern of occurrence of bio-duck call types to unravel if phrases occur in repeating patterns that can be identified as themes. Given that challenges that this species is and will be facing in the context of retreating sea ice and increased occurrence of climate anomalies, systematic monitoring and processing of AMW vocal behavior may help to provide prompt answers to understand how we can effectively conserve this species in its critical habitat.

## Supplementary Information

Supplementary Information.

## Data Availability

Data availability Analyses reported in this article can be reproduced using the data provided: https://datadryad.org/stash/share/NH1Pfm-asso8lEGwMfvCw8ZW0y1ILbfZS1ApM5NEBGA. Dataset generated during this study is also available from corresponding author upon reasonable request. Data used in study is public. Data were collected under Umweltbundesamt (UBA) permits no. I 3.5-94003-3/286 (Expedition ANT-XXIX/2), II 2.8-94003-3/324 (Expedition ANT-XXX/2), and II 2.8-94003-3/38 (Expedition PS103).
