# Supplementary material for: Spatial and temporal variability of the acoustic repertoire of Antarctic minke whales (Balaenoptera bonaerensis) in the Weddell Sea
Source: Sci Rep. 2023 Jul 22;13:11861. doi: 10.1038/s41598-023-38793-4 (PMC10363173; doi:10.1038/s41598-023-38793-4)
Supplement: Supplementary file 1 — Supplementary Information. [file 41598_2023_38793_MOESM1_ESM.docx]

**Spatial and temporal variability of the acoustic repertoire of Antarctic minke whales (*Balaenoptera bonaerensis*) in the Weddell Sea.**

*Diego Filún ﻿^1,2^, Ilse van Opzeeland ^1,3^

1. **﻿**Ocean Acoustics Lab, Alfred-Wegener-Institute Helmholtz-Zentrum für Polar- und Meeresforschung, 27570 Bremerhaven, Germany
2. Centro FONDAP- de Investigación en Dinámica de Ecosistemas Marinos de Altas Latitudes (IDEAL), Valdivia, Chile
3. ﻿Helmholtz Institute for Functional Marine Biodiversity (HIFMB), Carl von Ossietzky University, 26129 Oldenburg, Germany

*Contact information: [diego.filun@awi.de](mailto:diego.filun@awi.de)

# Appendix

Appendix Figure 1. Left figure (A) showing a spectrogram and waveform of bio-duck call type A2. Right spectrogram (B) shows the different measurements that were automatically extracted. Numbers in red correspond to the length in seconds of the different measurements (Table. 2). The example represents an SNR= 12 dB and a threshold = 20%.

Appendix Table 1. Description of the variables automatically measured to classify the different Bio-duck call types

| **NP** | Number of pulses | N° of pulses that form a Bio-duck call |
| --- | --- | --- |
| **TD** | Total duration | Time length between the start time of the first pulse detected in the Bio-duck sequence until the time when finish the last pulse detected in the sequence who compose a call |
| **DFP** | Duration first pulse | Time duration of the first pulse that forms a Bio-duck call |
| **DLP** | Duration last pulse | Time duration of the last pulse that forms a Bio-duck call |
| **IPI** | mean inter-pulse interval | Mean of the time duration of all the inter-pulse intervals in a Bio-duck call |
| **PF** | Peak frequency | Frequency of maximum power value obtained measuring the down sweep component of the Bio-duck calls |

Appendix Table 2. Classification and measured values obtained of the different Bio-duck calls identified (*n*=60 for every Bio-duck call type). Calls with * were detected only in PALAOA

| **Group** | **N° Pulses** | **Total Duration (seconds)** | **1st Pulse (seconds)** | **Last Pulse (seconds)** | **Inter-pulse interval (seconds)** | **Peak Frequency (Hertz)** |
| --- | --- | --- | --- | --- | --- | --- |
| A* | 1 | 1.2 ± 0.3 | 1.2 ± 0.5 |  |  | 115 ± 12 |
| A | 2 | 1.7 ± 0.5 | 0.7 ± 02 | 0.4 ± 0.02 | 0.5 ± 0.003 | 114 ± 18 |
| B* | 4 | 1.4 ± 0.2 | 0.1 ± 0.04 | 0.1 ± 0.04 | 0.03 ± 0.002 | 194 ± 20 |
| B | 5 | 1.5 ± 0.2 | 0.1 ± 0.05 | 0.1 ± 0.03 | 0.03 ± 0.002 | 191 ± 22 |
| B | 6 | 1.4 ± 0.4 | 0.1 ± 0.04 | 0.06 ± 0.02 | 0.05 ± 0.004 | 197 ± 9 |
| B | 7 | 1.5 ± 0.4 | 0.1 ± 0.04 | 0.1 ± 0.02 | 0.04 ±0.002 | 186 ± 25 |
| B | 8 | 1.7 ± 0.4 | 0.1 ± 0.03 | 0.1 ± 0.01 | 0.04 ± 0.002 | 177 ± 22 |
| B | 9 | 1.9 ± 0.7 | 0.1 ± 0.05 | 0.08 ± 0.05 | 0.03 ± 0.001 | 184 ± 18 |
| C* | 2 | 1.4 ± 0.6 | 0.2 ± 0.03 | 0.1 ± 0.04 | 0.09 ± 0.002 | 134 ± 11 |
| C | 3 | 1 ± 0.4 | 0.2 ± 0.01 | 0.2 ± 0.06 | 0.2 ± 0.02 | 138 ± 14 |
| C | 4 | 1.4 ± 0.6 | 0.3 ± 0.06 | 0.2 ± 0.05 | 0.1 ± 0.01 | 145 ± 20 |
| C | 5 | 1.6 ± 0.3 | 0.2 ± 0.06 | 0.1 ± 0.03 | 0.09 ± 0.002 | 135 ± 26 |
| C | 6 | 1.8 ± 0.3 | 0.1 ± 0.02 | 0.1 ± 0.02 | 0.04 ± 0.01 | 138 ± 33 |
| D* | 3 | 1.2 ± 0.5 | 0.1 ± 0.01 | 0.2 ± 0.03 | 0.2 ± 0.02 | 145 ± 28 |
| D | 4 | 1.2 ± 0.5 | 0.2 ± 0.01 | 0.1 ± 0.02 | 0.2 ± 0.02 | 142 ± 19 |
| D | 5 | 1.5 ± 0.3 | 0.2 ± 0.01 | 0.1 ± 0.02 | 0.1 ± 0.01 | 152 ± 21 |

Appendix Table 3. Percentage of occurrence of the different Bio-duck types for every location. Monitoring sites HAFOS and PALAOA

| Area | Positions-Data | A1 | A2 | B4 | B5 | B6 | B7 | B8 | B9 | C2 | C3 | C4 | C5 | C7 | D3 | D4 | D5 |
| --- | --- | --- | --- | --- | --- | --- | --- | --- | --- | --- | --- | --- | --- | --- | --- | --- | --- |
| HAFOS | AWI251-1008 | - | 27.4 | - | - | - | - | 6.5 | - | - | 2.5 | 4.0 | 0.5 | - | - | 59.2 | - |
|  | AWI207-1034 | - | 37.3 | - | - | - | 1.5 | 9.0 | 3.6 | - | 2.3 | 1.2 | 0.1 | - | - | 43.5 | 1.6 |
|  | AWI208-1030 | - | 61.9 | - | - | - | 1.3 | 3.7 | 0.1 | - | 1.1 | 0.5 | 0.4 | - | - | 19.2 | 11.8 |
|  | AWI249-1014 | - | 59.3 | - | - | 0.6 | 3.2 | - | 3.8 | - | 0.4 | 1.5 | - | - | - | 24.1 | 7.2 |
|  | AWI248-1013 | - | 54.8 | - | - | - | - | 12.7 | 1.4 | - | 1.5 | 2.8 | - | - | - | 7.1 | 19.7 |
|  | AWI232-1011 | - | 49.1 | - | - | 1.4 | 4.8 | 1.2 | 15.4 | - | 2.5 | 0.6 | 1.0 | 0.1 | - | 1.7 | 22.3 |
| PALAOA | 2015.0 | 8.7 | - | 18.7 | 47.4 | - | - | - | - | 8.0 | - | 14.8 | - | - | - | - | 2.4 |
|  | 2016.0 | 42.8 | - | 2.0 | 21.1 | 1.0 | - | - | - | 10.7 | 15.6 | - | - | - | 3.7 | 3.1 | - |
|  | 2017.0 | - | 18.8 | - | - | 1.4 | 4.7 | 1.2 | 15.4 | - | 32.7 | 0.6 | 1.0 | 0.1 | - | 1.7 | 22.3 |


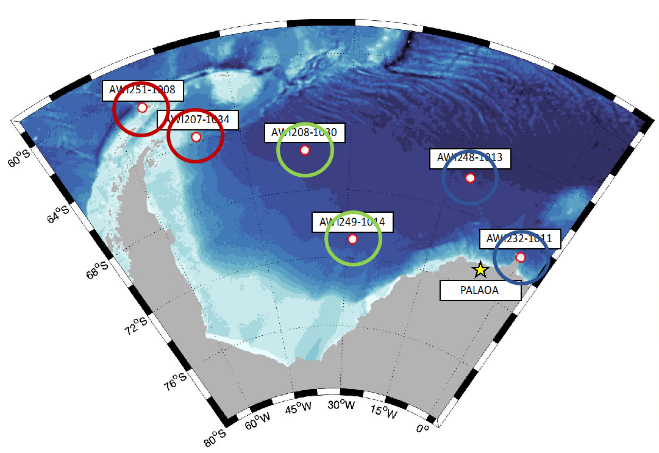


Appendix Figure 2. Dendrogram based on the acoustic repertoire per mooring position across the Weddell Sea. Dots in red represent clusters with AU larger than 95%.

We used the variability of calls recorded at each monitoring site to perform an agglomerative hierarchical cluster analysis to generate groups of unsupervised unit types. A Euclidean method was used to calculate the distances between the different clusters. To determine the number of clusters (k), the package 30 "NbClust" was used. It automatically calculates and provides 30 different indices to determine an appropriate number of k from the different results obtained by varying all combinations for the number of clusters, distance measures and clustering methods.

The dendrogram allows to clearly appreciate the clustering relationships between monitoring sites, based on the type of calls recorded at each of those locations.

The unsupervised analysis agglomerated the different monitoring positions into three groups.

The first group agglomerated positions AWI251-1008 and AWI207-1034 (red), the second group agglomerated positions AWI208-1030 and AWI249-1014 (green). The third and last group is composed of the positions AWI248-1013 and AWI232-1011 (blue) (Appendix Figure 2).

The generation of the clusters coincides in how the monitoring sites are distributed throughout the Weddell Sea.

The red cluster corresponds to the positions located close to the Antarctic Peninsula (West Weddell Sea), the green cluster corresponds to the sites in the center of the Weddell Sea and the blue cluster corresponds to the positions located in the East Weddell Sea.

In addition, the positions in the central Weddell Sea are more similar to those on the west side of the Weddell Sea than those near the Antarctic Peninsula.

In conclusion, the acoustic repertoire of Antarctic minke whales in the Weddell Sea is not homogeneous, but there is variability between sites. Based on our data, there are three groups with different acoustic repertoire in the study area, suggesting that there are three acoustically distinct groups in the Weddell Sea or that the variability in acoustic repertoire may be due to differences in habitat use by Antarctic minke whales throughout the Weddell Sea. Further description and justification of the hypotheses postulated for these results is developed in the Discussion and Conclusions section of the manuscript.


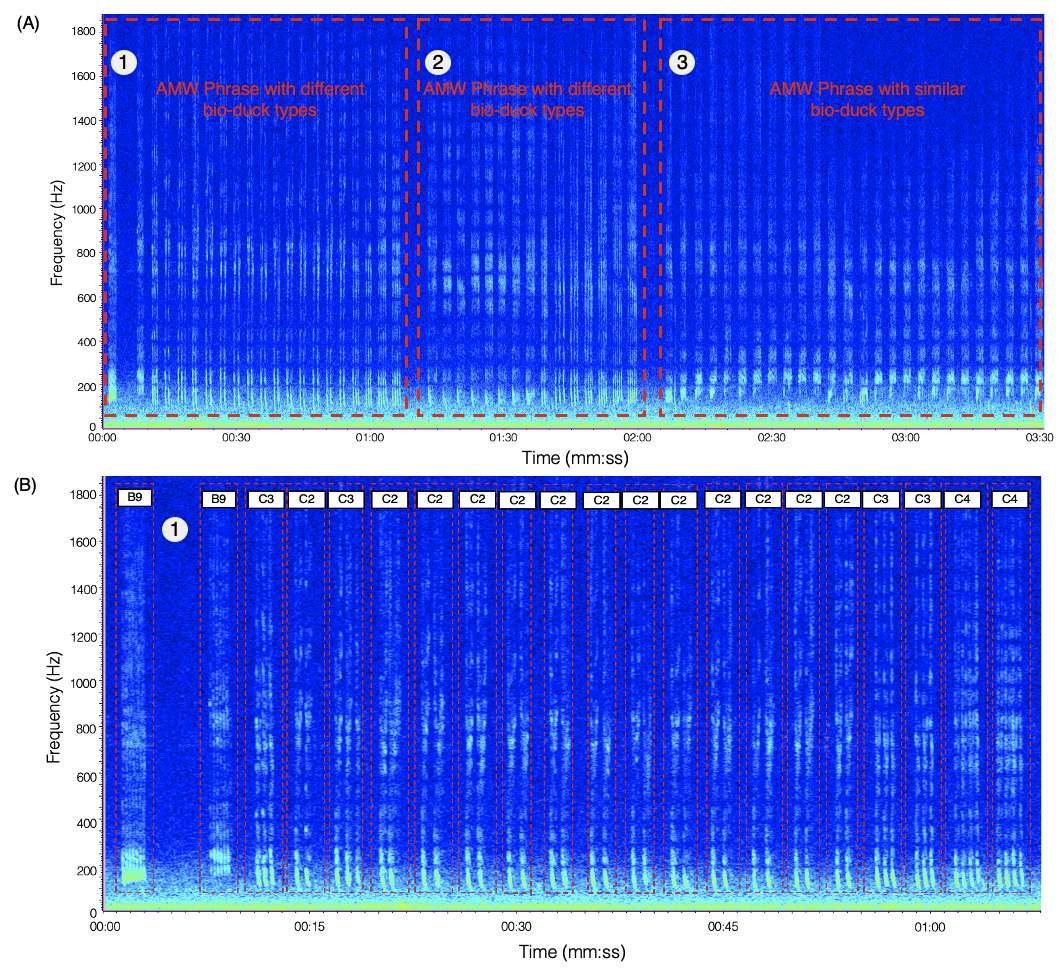
Appendix Figure 3. (A) Spectrogram showing three different phrases of AMWs song (1) AWM phrase composed with different bio-duck types, (2) AMW phrase composed with different bio-duck types and (3) AMW phrase with similar bio-duck types. (B) Zoomed spectrogram of phrase 1 in which the different types of bio-duck units that form the potential phrase can be identified (unit types listed above red dashed selection boxes).


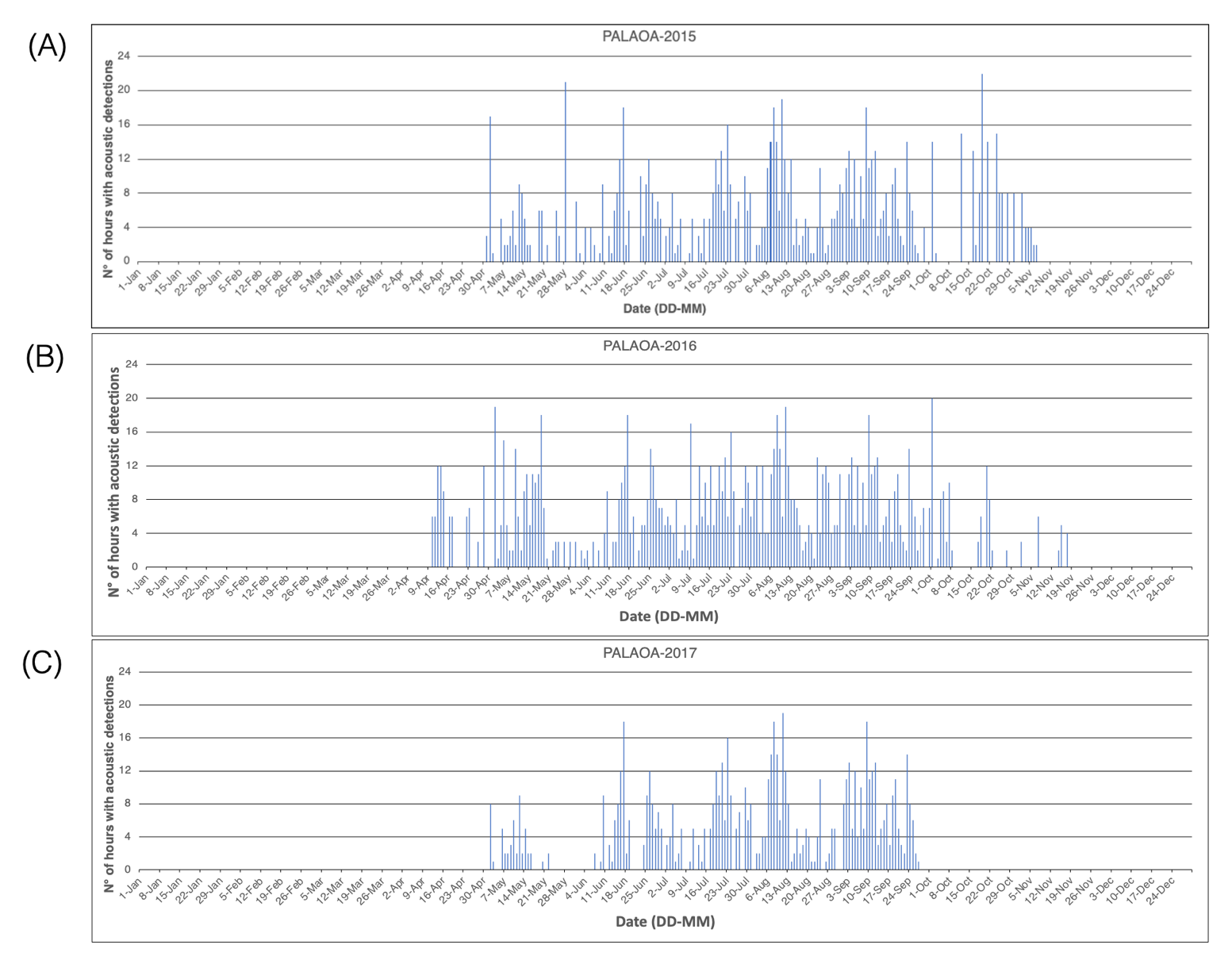


Appendix Figure 4. (A) Seasonal occurrence of AMW bio-duck calls during 2015. (B) Seasonal occurrence of AMW bio-duck calls during 2016. (C) Seasonal occurrence of AMW bio-duck calls during 2017.
